# Potential for Co-Infection of a Mosquito-Specific Flavivirus, Nhumirim Virus, to Block West Nile Virus Transmission in Mosquitoes

**DOI:** 10.3390/v7112911

**Published:** 2015-11-11

**Authors:** Silvina Goenaga, Joan L. Kenney, Nisha K. Duggal, Mark Delorey, Gregory D. Ebel, Bo Zhang, Silvana C. Levis, Delia A. Enria, Aaron C. Brault

**Affiliations:** 1 Instituto Nacional de Enfermedades Virales Humanas, Pergamino 2700, Argentina; silvigoenaga@gmail.com (S.G.); slevis0@yahoo.com (S.C.L.); deliaenria@anlis.gov.ar (D.A.E.); 2 Division of Vector-Borne Diseases, Centers for Disease Control and Prevention, Fort Collins, CO 80521, USA; vwx1@cdc.gov (J.L.K.); wwd3@cdc.gov (N.K.D.); esy7@cdc.gov (M.D.); 3 Department of Microbiology, Immunology and Pathology, Colorado State University, Fort Collins, CO 80523, USA; Gregory.Ebel@colostate.edu; 4 Wuhan Institute of Virology, Chinese Academy of Sciences, Wuhan, Hubei 430071, China; zhangbo@wh.iov.cn

**Keywords:** superinfection exclusion, mosquito, inhibition, barrier, transmission

## Abstract

Nhumirim virus (NHUV) is an insect-specific virus that phylogenetically affiliates with dual-host mosquito-borne flaviviruses. Previous *in vitro* co-infection experiments demonstrated prior or concurrent infection of *Aedes albopictus* C6/36 mosquito cells with NHUV resulted in a 10,000-fold reduction in viral production of West Nile virus (WNV). This interference between WNV and NHUV was observed herein in an additional *Ae. albopictus* mosquito cell line, C7-10. A WNV 2K peptide (V9M) mutant capable of superinfection with a pre-established WNV infection demonstrated a comparable level of interference from NHUV as the parental WNV strain in C6/36 and C7-10 cells. *Culex quinquefasciatus* and *Culex pipiens* mosquitoes intrathoracically inoculated with NHUV and WNV, or solely with WNV as a control, were allowed to extrinsically incubate the viruses up to nine and 14 days, respectively, and transmissibility and replication of WNV was determined. The proportion of *Cx. quinquefasciatus* mosquitoes capable of transmitting WNV was significantly lower for the WNV/NHUV group than the WNV control at seven and nine days post inoculation (dpi), while no differences were observed in the *Cx. pipiens* inoculation group. By dpi nine, a 40% reduction in transmissibility in mosquitoes from the dual inoculation group was observed compared to the WNV-only control. These data indicate the potential that infection of some *Culex* spp. vectors with NHUV could serve as a barrier for efficient transmissibility of flaviviruses associated with human disease.

## 1. Introduction

The genus *Flavivirus* (family Flaviviridae) is comprised of 53 virus species of enveloped, single-stranded, positive-sense RNA viruses. Flaviviral genomes are approximately 11 kb in length and encode a single long open-reading frame that is cleaved into three structural (C, prM, E), and seven non-structural (NS1, NS2A, NS2B, NS3, NS4A, NS4B, and NS5) proteins and two known protein variants (NS1′ and 2K peptide) [1,2,3]. Phylogenetic analyses of flaviviruses have demonstrated clustering based on host preference range: insect-specific flaviviruses (ISFs), dual-host tick-borne flaviviruses (TBFVs), viruses with no known vector (NKV), or mosquito-borne (dual-host) flaviviruses (MBFVs) [4,5]. However, a number of recent viruses have been described whose sequences cluster with the MBFVs phylogenetically but have an apparent insect-specific host restrictive phenotype. These viruses have tentatively been designated as “Unidentified Vertebrate Host Flaviviruses” (UVHF) [6] and include representatives from wide geographic locations: Chaoyang virus (CHAOV) from the Republic of Korea [7] and China [8], Donggang virus (DONV) from China [9], Barkedji virus from Senegal and Israel [10], Nounane virus (NOUV) from Côte d’Ivoire [11], Lammi (LAMV) and Ilomantsi (ILOV) viruses from Finland [12,13], Nanay virus from Peru [14], Marisma mosquito virus (MMV) from Spain [15] and Italy [16], and Nhumirim virus (NHUV) from Brazil [6,17]. The preponderance of data suggests that these viruses are not capable of infecting vertebrate cells and the use of the term “Unidentified Vertebrate Host” has been chosen to serve as an antithetical term to “No Known Vector” for flaviviruses that have been described that have not demonstrated a capacity to replicate in invertebrate cells.

All described flaviviruses associated with human disease fall within the MBFV and TBFV host range groups including dengue virus (DENV1-4) [18], yellow fever virus (YFV), Japanese encephalitis virus (JEV), West Nile virus (WNV) [19], St. Louis encephalitis virus (SLEV) [20], and tick-borne encephalitis virus (TBEV) [21]. These viruses cause millions of human infections each year ranging from mild febrile symptoms to fatal hemorrhagic/neurologic disease. The fact that UVHFs and ISFs often infect the same mosquito vectors used by dual host MBFs for transmission of these agents to humans and the relative genetic similarity between these flaviviruses led to the independent assessment by many research groups of the potential for replicative interference between ISFs and MBFs; however, despite the positive association between genetic relatedness of the infecting and superinfecting virus for the efficacy of superinfection exclusion (SIE) [22], only one evaluation of interference has been reported between a UVHF and MBFV [6].

Superinfection studies with MBFVs and ISFs have been performed in order to establish a preventative intervention strategy for blocking the transmission of agents of human diseases and in order to gain a better understanding of any additional factor(s) that could alter vector competence of mosquitoes in both enzootic and epizootic transmission cycles. A number of studies have assessed the relative potential of different ISFs [Culex flavivirus (CxFV) and Palm Creek virus (PCV)] to interfere with replication of flaviviruses of human health importance in cultured mosquito cells. The *in vitro* interference between an ISF and WNV was supported by a study in which C6/36 cells previously inoculated with a CxFV from Colorado demonstrated reduced WNV titers at early time points post infection [23]. Previous inoculation of C6/36 cells with PCV was found to have a significant effect on replication of both WNV and Murray Valley encephalitis virus [24]. In contrast, Kent *et al.* demonstrated that pre-inoculation of C6/36 cells with CxFV from Guatemala had no interfering effect on subsequent WNV replication [25], and Kuwata *et al.* observed no evidence of SIE of JEV and DENV2 derived from *Culex tritaeniorhynchus* cells persistently infected with a Japanese strain of CxFV [26]. Variable interference observed for WNV replication with prior infection of mosquito cells with PCV and CxFV could very likely be due to genetic differences between PCV and CxFV viruses or the WNV challenge (lineage 1a *vs.* 1b) viruses utilized.

Few *in vivo* studies have addressed the importance of superinfection of MBFVs with ISFs, and no *in vivo* assessments of cross-interference have been reported between MBFVs and UVHFs. Two studies have directly addressed the potential for SIE between CxFV and WNV. In one study, performed with colonized *Culex pipiens* mosquitoes persistently infected with CxFV, a significantly lower dissemination rate with WNV was observed at days post-inoculation (dpi) seven; however, differences in the infection and transmission rates were not detected [23]. In a study performed with sequentially infected *Culex quinquefasciatus*, no differences in infection, dissemination, or transmission were observed. Interestingly, when these mosquitoes were co-inoculated with CxFV and WNV, a higher percentage of mosquitoes were observed to transmit WNV while no difference was observed in the other mosquito colony [25]. These *in vivo* studies indicate a definite potential for the positive and negative modulation of vector competence for a flavivirus of human health significance that is likely ISF virus- and mosquito strain-dependent.

Previous *in vitro* experiments have demonstrated robust inhibition of WNV, SLEV, and JEV in mosquito cells previously or concurrently infected with the UVHF virus, NHUV [6]. In order to assess the potential that UVHFs, more closely genetically related to MBFVs than ISFs, have a greater potential for SIE, *Cx. quinquefasciatus* and *Cx. pipiens* mosquitoes were co-inoculated with NHUV and WNV, and the relative capacity for transmission compared to WNV-only inoculated mosquitoes was compared. Results described herein indicate the potential for *in vivo* SIE that results in reduced transmissibility of WNV. These data indicated the potential modulatory effect of certain pre-existing flaviviruses on the capacity for establishment of WNV superinfection in mosquitoes and highlights a potential method for blocking mosquito infection as a public health measure.

## 2. Material and Methods

### 2.1. In Vitro Assessment of SIE

In order to assess the *in vitro* SIE potential of NHUV for WNV in multiple mosquito cell lines, triplicate cultures of *Aedes albopictus* C6/36 and C7-10 cells were inoculated at an MOI of one with NHUV at five, three, one, or zero days prior to WNV inoculation (NY99 strain) at an MOI of 0.1 as previously described [6]. In order to control for cell density, all cell monolayers of C6/36 and C7-10 cells were prepared at the same time and density in 12-well plates. Subsequent WNV inoculations were performed at the designated times and titers compared to NHUV uninfected controls plates. Additionally, in order to assess the potential for previously described mutations to reduce the effect of NHUV SIE on WNV replication, a WNV mutant containing a point mutation (V9M) in the 2K peptide previously associated with increased replication in the presence of superinfecting WNV *in vitro* [22] and *in vivo* [27] was assessed for its capacity to replicate in the presence of superinfecting NHUV. These experiments were performed identically as those described in C6/36 and C7-10 cells for the wild type WNV strain.

### 2.2. Experimental Infection of NHUV in Cx. pipiens and Cx. quinquefasciatus

NHUV was originally isolated from *Cx. chidesteri* mosquitoes and, thus, was initially assessed for its relative competence to replicate in *Culex* spp. mosquitoes, *Cx. pipiens* (Chicago), and *Cx. quinquefasciatus* (Sebring), following intrathoracic (IT) inoculation. Approximately 3–6 day post-emergence, female *Cx. quinquefasciatus* and *Cx. pipiens* were inoculated with 9.1 log_10_ TCID_50_/mL NHUV diluted 1:10 in a solution of Dulbecco’s modified Eagle medium (DMEM). A separate group of mosquitoes reared concurrently from the same colony were inoculated with DMEM only, serving as inoculation controls. Inoculated mosquitoes were housed in screened paperboard pint containers and held at 28 °C and 95% relative humidity (RH) with 16:8 L/D lighting cycles. At seven days post-inoculation, bodies, legs, and saliva were harvested from each mosquito. Saliva was collected by insertion of the proboscis of the mosquito in a capillary tube charged with immersion oil as previously described [28]. Briefly, mosquitoes were anesthetized by Carolina FlyNap^®^ (triethylamine) and the proboscis inserted into a capillary tube containing 5 µL Cargill Type B immersion oil. After 30 min of salivation, the mosquitoes’ probosces were removed from the capillary tubes, and legs and bodies separated into individual tubes. Each capillary tube containing salivary expectorate was expelled from the capillary into 300 µL of DMEM with 20% fetal bovine serum (FBS), 1% l-glutamine, 1% Fungizone, and 1% penicillin streptomycin. Bodies and legs were each homogenized separately in microcentrifuge tubes containing a single copper BB and 1 mL DMEM with 20% FBS, 1% l-glutamine, 1% Fungizone and 1% penicillin streptomycin. Mosquitoes were ground for 4 min at 20 cycles per second on a mixer mill MM300 (Retsch, Haan, Germany). Homogenates were clarified by centrifugation at 5000× *g* for 10 min at 4 °C. Supernatants were stored at −80 °C until assayed for NHUV antigen or RNA (described below).

### 2.3. RT-PCR and Immunofluorescence Detection of NHUV Infection

Monolayers of C6/36 cells were inoculated with 50 µL of supernatant from clarified homogenates of mosquito bodies or from salivary expectorants. Supernatants were harvested or cells fixed with an acetone/phosphate buffered saline (PBS) solution (3:1) for 1 h three days post-infection (dpi) for reverse-transcription polymerase chain reaction (RT-PCR) amplification or performance of immunofluorescence assays (IFA), respectively. RNA was extracted using QIAamp Viral Minikit (Qiagen, Germantown, MD, USA) prior to RT-PCR with Qiagen one-step RT-PCR kit using flavivirus universal primers [29]. Amplicons were visualized by gel electrophoresis. Immunofluorescence assays (IFA) using monoclonal flavivirus antibody (Mab4G2) were utilized to evaluate NHUV infection of the inoculated/fixed C6/36 cells.

### 2.4. Vertical Transmission Assessment

Groups of *Cx. pipiens* Chicago strain mosquitoes were intrathoracically inoculated with NHUV as described above. These NHUV-inoculated mosquitoes were sugar-starved 24 h prior to being offered an oral blood meal with defibrinated calf blood (Colorado Serum Company, Denver, CO, USA) five days after intrathoracic inoculation. Mosquitoes were allowed to feed for 30 min from a Hemotek feeder (Discovery Workshops, Accrington, UK). After that period, mosquitoes were cold anesthetized, and all the non-engorged females were removed from the carton. The remaining engorged females were placed individually in 15 mL glass test tubes and provided an oviposition substrate with free access to a 10% sucrose solution at 28 °C, 95% RH, and 16:8 (light:dark) photoperiod. All females that laid egg rafts were removed from the tubes, triturated as described above, and tested for the presence of NHUV RNA and infectious virus by RT-PCR and IFA, respectively as described above. The eggs obtained from NHUV-inoculated females were placed in pans with water and food, and the larvae were reared to the adult stage. All F1 emergent adult mosquitoes from those females that were found to be positive by IFA and/or RT-PCR for NHUV or NHUV RNA were processed. Bodies from F1 adults were individually assayed as described above.

### 2.5. Dual infection Vector Competence Assay

3–5 day post-emergence *Cx. quinquefasciatus* Sebring strain mosquitoes and *Cx. pipiens* Chicago strains were inoculated intrathoracically with NHUV as previously described. A control group of DMEM inoculated mosquitoes was included. Seven days post-infection oral blood feeds were performed as described above. Blood meals were prepared with defibrinated calf blood (Colorado Serum Company) at a final concentration of 8 log_10_ PFU/mL of WNV NY99. An aliquot of the blood was assayed for infectious units by plaque assay on Vero cells [30]. Survivorship in the sequential infection series was low, so additional assessments in which *Culex* spp. mosquitoes were co-inoculated with NHUV and WNV were performed. *Cx. pipiens* and *Cx. quinquefasciatus* mosquitoes were intrathoracically co-inoculated with approximately 1000 TCID_50_ units of NHUV and 100 PFU of WNV or solely with WNV as a control. *Cx. pipiens* mosquitoes were allowed to extrinsically incubate the viruses for 14 days and *Cx. quinquefasciatus* for three, five, seven, or nine days, at which point transmissibility and relative replication of the WNV determined. Bodies and saliva from each mosquito were processed and WNV infection rate (body) and WNV transmission rate (saliva) were analyzed by plaque titration on 12-well plates of Vero cells. The percentage of mosquitoes that were infected, demonstrated disseminated infections, and demonstrated theoretical transmission of WNV were compared by a Fisher exact test. The mean WNV titers in mosquito bodies and saliva at 14 dpi were compared between mosquitoes co-infected with NHUV + WNV or infected with WNV alone by ANOVA with a multiple comparison adjusted Type I error using Sidak’s method. A generalized linear model, assuming a binomial distribution, was fit to the proportions of mosquitoes that became infected and transmitted WNV. Confidence intervals were computed for odds ratios using Sidak’s method for multiple comparisons.

## 3. Results

### 3.1. In Vitro SIE Assessment

**Figure 1:**
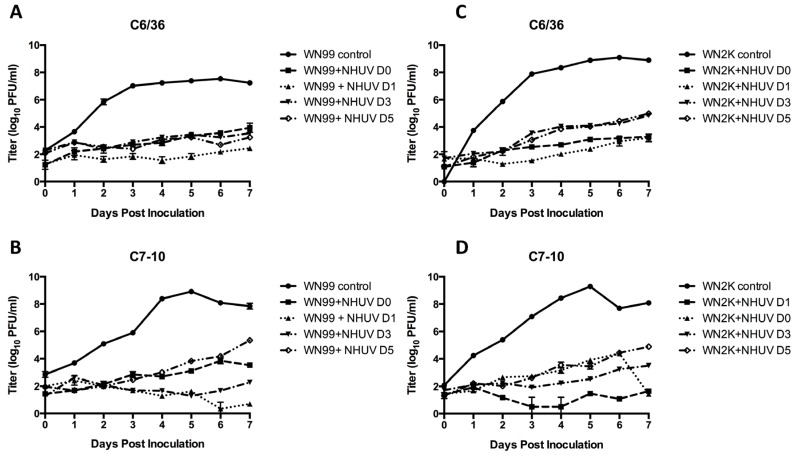
WNV titers obtained from culture supernatants of C6/36 (panels **A**,**C**) and C7-10 (panels **B**,**D**) cells inoculated with NHUV and superinfected with WNV (panels **A**,**B**) or the WNV V9M 2K mutant (panels **C**,**D**) at zero, one, three, and five dpi. Each data point represents the plaque titration mean of triplicate cultures. Bars represent standard deviations from the mean. The limit of detection for WNV titers was 5 PFU/mL culture supernatant.

In agreement with previous studies, C6/36 cells inoculated with NHUV demonstrated a marked reduction in WNV titers at all pre-incubation times (Figure 1A). Although the peak titer of WNV replication was approximately 25-fold higher in C7-10 cells (Figure 1B) than C6/36 for NHUV uninfected cells, the superinfection patterns were very consistent between the cell lines, with both C6/36 and C7-10 cells exhibiting a 4000-fold decrease in WNV in cells with pre-established NHUV infection (Figure 1A,B). Interestingly, the NS4A 2K peptide mutant grew to significantly higher titers than the wild type WNV strain in C6/36 cells, but no difference in SIE was evident for the 2K mutant (Figure 1A *vs.* Figure 1C). In fact, the inhibition of this mutant was more pronounced than that of the parental WNV in both cell lines, with at least 12,000 and 31,000-fold reductions in titers for C6/36 and C7-10 cells (Figure 1C *vs.* Figure 1D), respectively.

### 3.2. Experimental Infection of NHUV in Cx. pipiens and Cx. quinquefasciatus

**Figure 2:**
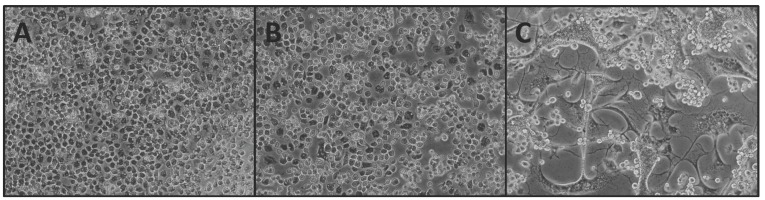
Image of C6/36 cells inoculated with homogenates from *Cx. quinquefasciatus* previously infected with NHUV by IT-inoculation. (**A**) Negative control (mock inoculation); (**B**) C6/36 cells inoculated with salivary expectorants from NHUV IT-inoculated *Cx. quinquefasciatus*; and (**C**) C6/36 inoculated with homogenate from the body of a *Cx. quinquefasciatus* mosquito demonstrating syncytia and rounded cells.

Experimental infections with NHUV by intrathoracic inoculation in *Cx. pipiens* and *Cx. quinquefasciatus* mosquitoes demonstrated productive viral replication in both species. C6/36 cells that were inoculated with salivary expectorants failed to elicit CPE in inoculated C6/36 cells (Figure 2B), despite having demonstrated positive signal by IFA and RT-PCR. C6/36 cells inoculated with body homogenates and salivary expectorants from IT-inoculated mosquitoes were positive for NHUV by immunofluorescence assay and by RT-PCR. Cytopathic effects (CPE) were observed in C6/36 cells inoculated with homogenates from bodies in both species after three dpi (*Cx. quinquefasciatus*; Figure 2C).

### 3.3. Vertical Transmission Assay

Evidence of vertical transmission of NHUV in *Cx. pipiens* mosquitoes following intrathoracic inoculation was demonstrated. Sixty-eight *Cx. pipiens* females were IT-inoculated with NHUV; however, offspring were reared to adults (*n* = 3) from only one NHUV IT-inoculated female that successfully oviposited. Of these, one mosquito was positive for NHUV by RT-PCR and by IFA after homogenization and inoculation on C6/36 cells.

### 3.4. Co-Infection of NHUV/WNV in Cx. pipiens mosquitoes

In order to assess the potential inhibitory effect of NHUV replication on WNV infectivity and transmissibility, *Cx. pipiens* mosquitoes were intrathoracically co-inoculated with NHUV and WNV in a 10:1 ratio or solely with WNV as a control. Mosquitoes co-inoculated with NHUV + WNV (*n* = 33) were processed at 14 dpi. Transmission, dissemination and infection rates were analyzed (Table 1). WNV infection and dissemination rates were 100% for both groups (co-infected and control mosquitoes). The transmission rate in the NHUV + WNV group (45.5%, *n* = 33) was not significantly different from those inoculated with WNV alone (54.6% *n* = 11) (Table 1). Next, WNV titers were compared between the experimental and control groups at 14 dpi (Table 2). The average titer for WNV in bodies of co-inoculated mosquitoes was not significantly different than the WNV-only control group (5.8 log_10_ PFU/mosquito and 6.2 log_10_ PFU/mosquito, respectively). Similarly, the average dissemination titers were not significantly different between groups (2.4 and 2.5 log_10_ PFU/mosquito for the dual and single infection groups, respectively). WNV titers in the saliva were also not significantly different between control and the dual infection groups (0.8 and 0.6 log_10_ PFU/mosquito, respectively).

**Table 1:** WNV infection and transmission rates of NHUV co-inoculated *Cx. pipiens* mosquitoes at 14 dpi.

Treatment Group	Infection (%)	Transmission (%)
NHUV + WNV	33/33 (100)	15/33 (45.5)
Sham + WNV	11/11 (100)	6/11 (54.6)

**Table 2:** WNV titers in bodies, legs and saliva of NHUV co-inoculated *Cx. pipiens* mosquitoes at 14 dpi.

Treatment Group	Bodies (PFU/Mosquito)	Legs (PFU/Mosquito)	Saliva (PFU/Expectorant)
NHUV + WNV	5.8 ± 1.1 *	2.4 ± 0.7	0.6 ± 0.9
Sham + WNV	6.2 ± 0.2	2.5 ± 0.4	0.8 ± 1.0

* ± log_10_ standard deviation from the mean.

### 3.5. Co-Infection of NHUV/WNV in Cx. quinquefasciatus mosquitoes

The capacity of *Cx. quinquefasciatus* Sebring for being infected and transmitting WNV when co-inoculated simultaneously with NHUV was also assessed. Co-inoculated mosquitoes were harvested and processed at three, five, seven, and nine dpi. Results demonstrated 100% infection with WNV of the *Cx. quinquefasciatus* mosquitoes at all four time points in the control groups. Similarly, the NHUV + WNV experimental infection group exhibited 100% infection at dpi five, seven, and nine, with 91% infection in the co-inoculated group at dpi three (Table 3). With the exception of dpi three (*p* < 0.0001), no significant differences in the mean viral titer between the dual and control WNV inoculation groups were detected from triturated bodies (Table 4). Although there was an observed trend for the titers in saliva to be higher in the control compared to the dual infection groups, no significant differences were determined. Nevertheless, differences in the proportion of mosquitoes that were capable of transmitting WNV (≥21 mosquitoes per sampling time point) were significantly lower for the NHUV + WNV group than the WNV control at dpi seven and nine (Table 3) [6.3 odds ratio (95% CI 1.4 to 27.8) for WNV *vs.* NHUV + WNV at dpi seven and 6.4 odds ratio (95% CI 1.3 to 31.5) for WNV *vs.* NHUV + WNV at dpi nine]. Analyses of the mean WNV titer in expectorants obtained from the control *vs.* dual infection groups showed no significant differences at any of the time points.

**Table 3:** WNV infection and transmission rates of NHUV co-inoculated *Cx. quinquefasciatus* mosquitoes at three, five, seven, and nine dpi.

Sampling	Treatment Group	Infection Rate (%)	Transmission Rate (%)
3 dpi	NHUV + WNV	19/21 (91)	0/21 (0)
	WNV	32/32 (100)	7/32 (21.8)
5 dpi	NHUV + WNV	38/38 (100)	19/38 (50)
	WNV	32/32 (100)	11/32 (34)
7 dpi	NHUV + WNV	30/30 (100)	10/30 (33.3)
	WNV	25/25 (100)	19/25 (76) *
9 dpi	NHUV + WNV	21/21 (100)	5/21 (23.8)
	WNV	27/27 (100)	18/27 (66.7) *

* Indicates significantly higher transmission rates for WNV infection group *vs.* the dual inoculation group.

**Table 4:** WNV titers in bodies and saliva of NHUV co-inoculated *Cx. quinquefasciatus* mosquitoes at three, five, seven, and nine dpi.

Sampling	Treatment Group	Bodies (PFU/Mosquito) *	Saliva (PFU/Expectorant) *
3 dpi	NHUV + WNV	5.2 ± 1.9	ND ^†^
	WNV	6.5 ± 1.1	0.3 ± 0.6
5 dpi	NHUV + WNV	6.7 ± 0.5	1.1 ± 1.3
	WNV	6.4 ± 0.5	0.7 ± 1.1
7 dpi	NHUV + WNV	6.2 ± 0.4	0.8 ± 0.2
	WNV	6.2 ± 0.4	2.0 ± 1.4
9 dpi	NHUV + WNV	6.0 ± 0.3	0.4 ± 0.9
	WNV	6.2 ± 0.3	1.8 ± 1.6

* log_10_ standard deviation from the mean; ^†^ ND: not done.

## 4. Discussion

Data presented herein indicate productive infection and subsequent oral transmission of NHUV following intrathoracic inoculation in *Cx. pipiens* and *Cx. quinquefasciatus* mosquitoes; however, the relevance of the transmissibility of NHUV orally by salivation has yet to be determined in the context of its potential insect-specific host restrictive phenotype [6]. However, the observation of NHUV in the saliva of inoculated *Culex* spp. mosquitoes indicates that SIE mechanisms could be critical for blocking successful transmission of other flaviviruses at all stages of extrinsic incubation including the infection and escape from the salivary glands.

Attempts to grow NHUV in limited vertebrate cell lines have proven unsuccessful [6,17]. The phylogenetic placement of NHUV with other UVHF viruses in close proximity to MBFs, coupled with the similar codon usage pattern of NHUV to MBFs, indicate that if the vertebrate host-restricted phenotype does limit replication of UVHFs to mosquitoes, this restriction could have occurred relatively recently [6,9]. Despite a small sample size, vertical transmission of NHUV in F1 progeny of IT-inoculated female *Culex* spp. mosquitoes was demonstrated herein. This was in direct contrast to studies of an ISF, CxFV, in which an IT-inoculated colony of *Cx. pipiens* females, despite demonstrating viral RNA presence in the ovaries, failed to pass virus vertically. Interestingly, field-collected mosquitoes reared from isofemale lines that were found to be positive for CxFV were found to exhibit extremely high vertical infection and filial infection rates in their progeny [31]. While the assessment of the mechanisms and efficiency of vertical transmission of NHUV should be repeated on a much larger scale, it does suggest that this virus uses a maintenance method demonstrated by many classical ISFs [23,31,32,33].

Vector competence studies for WNV in *Cx. quinquefasciatus* mosquitoes co-infected with NHUV were performed and described herein. The results demonstrating significant reduction in the transmissibility of WNV in *Culex* mosquitoes co-inoculated with NHUV at later, rather than earlier, time points is interesting and portends that prior infection of mosquitoes with NHUV and an establishment of SIE mechanisms in salivary acinar cells critical for viral egress prior to exposure to WNV could result in an even more striking inhibition of WNV transmissibility. This notion is supported by the previous report that longer time intervals between initial infection and superinfection is critical for the magnitude of the inhibitory effects of SIE between DENV2 and DENV4 *in vitro* [34]. Future studies focused on assessing serial infections and monitoring the relative replication profiles of both viruses in dually infected mosquitoes will be critical to address this as a potential factor altering vector competence of field mosquito populations.

Superinfection barriers have been described previously for numerous arthropod-borne viruses in arthropods (mosquitoes, ticks and culicoides) as well as in arthropod cells [35,36,37,38,39,40,41]. For example, *Aedes triseriatus* mosquitoes experimentally infected with LaCrosse virus have been shown to be resistant to infection with another closely-related bunyavirus, Snowshoe Hare virus, after approximately two days post-infection [35]. Dual infections with Thogoto virus have been shown to establish resistance to co-infection for periods ranging between 1–10 days after the initial infection [36]. These data could indicate that the retarded replication rate of secondary flaviviral infections could be the result of pre-established monopolization of cellular transcriptional and translational machinery or the competitive blockage of cellular receptors. Replicating WNV replicons in BHK cells superinfected with WNV and other flaviviruses have demonstrated that preoccupation of intracellular resources such as host transcriptional and/or translational complexes are a likely explanation for the inhibition of the secondarily infecting virus [22]. It is interesting to note that despite previous studies that have demonstrated increased replication of the NS4A 2K peptide mutant (V9M) in the presence of a previous WNV infection both *in vitro* [22] and *in vivo* in mosquitoes [27], no such moderation of the effects of SIE were observed with this mutant when the initial infecting virus was NHUV. The 2K mutant WNV was capable of replicating to approximately 100-fold higher titers in C6/36 cells than the wild type WNV strain, while no difference in viral growth was observed between the two viruses in C7-10 cells. This finding likely indicates that the *in vitro* selection for this mutant capable of increased replicative efficiency at the RNA transcriptional level occurred in a cell-specific manner, afforded in the context of a homologous rather than heterologous superinfection.

The RNAi response in mosquitoes can serve as a significant selection pressure for diversification of viral genomes. WNV has been shown to incorporate mutations in its genome in regions of the viral genome commonly targeted by the RNAi response [42]. High genetic identity between NHUV and WNV in these critical highly-targeted regions of the genome could facilitate the secondary inhibition observed in the studies presented herein; however, the finding that NHUV infection in C6/36 cells that lack a functional RNAi response [43] demonstrated a robust SIE effect on WNV independent of NHUV infection timing compared to WNV infection [6] indicates that *in vitro* RNAi-mediated inhibition, at least in the case presented in this study, is likely not a mechanism of SIE. Enhanced viral growth of JEV and DENV2 was previously observed in *Cx. tritaeniorhynchus* cells persistently infected with CxFV, indicating the potential that previous infection with heterologous ISFs could potentially enhance infection by secondary MBFs [26]. An ecological association for this phenomenon was also represented by the finding of Newman *et al.* reported in 2011 in which a significant positive association between WNV and CxFV infection in individual female mosquitoes was observed [32]. One potential explanation for this phenomenon of secondary enhancement could be the suppression of an RNAi response by the initial ISF infection. The lack of enhanced WNV replication in RNAi-deficient C6/36 cells previously infected with CxFV [23], contrasted by the finding that *Cx. quinquefasciatus* inoculated with CxFV from Honduras exhibited higher WNV infection rates than non-CxFV inoculated mosquitoes [25], supports this possible explanation.

Several potential mechanisms could explain the lack of heterologous agent exclusion observed between MBFs and ISFs compared to those observed herein between a MBF and UVHF: (i) competitive exclusion through the scavenging of dissimilar genetic templates is inefficient; (ii) poorly-conserved amino acid identity between these viruses could reduce interfering heterologous protein-protein interactions; (iii) recombination events that result in non-functional products during RNA replication are infrequent due to poor efficiency of template switching; (iv) codon usage patterns between ISFs and MBFs are dissimilar enough that availability of tRNAs is not an impediment to translational efficiency of the secondary infecting virus; and (v) alternative intracellular trafficking could result in reduced direct interactions between the competing viruses. In contrast, the much higher degree of genetic identity between NHUV and WNV (54.3% nt, 49.4% aa) *vs.* between ISFs and WNV (34%–38% nt, 21%–26% aa) as well as the similar codon usage pattern [6] and the potentially recent acquisition of a mosquito restrictive phenotype for which intracellular trafficking of UVHFs much more closely resembles that of MBFVs could minimize these SIE impediments between WNV/NHUV and account for the more robust interference observed herein. Infection with genetically-distinctive secondary alphaviruses have demonstrated induction of SIE in C6/36 cells while unrelated bunyaviruses or flaviviruses could establish infection in Sindbis-infected C6/36 cells [37,39]. Furthermore, WNV replicon-expressing BHK cells were capable of blocking subsequent infection with a series of heterologous flaviviruses but were ineffectual in obstructing rhabdoviral or alphaviral superinfection [22]. The level of genetic identity necessary for effective induction of SIE has not been thoroughly established for any viral system nor has the specific mechanism(s) been determined. Synthetic biological approaches should allow for an efficient means to specifically address the relationship between the genetic identity of a superinfecting virus and the level of observed SIE. The close genetic identity between UVHFs and MBFVs could provide a foundation for robust inhibition that has not been recognized previously in MBFV and ISF interference experiments. Furthermore, UVHFs could serve as excellent research models for the direct assessment of the genetic basis for interference that could potentially be employed as additional intervention strategies for preventing mosquito transmission of human disease agents.
